# Managing Facial Palsy After Stroke: Results From an Online Survey of Health Professionals

**DOI:** 10.1111/1460-6984.70127

**Published:** 2025-09-16

**Authors:** Havva Sumeyye Eroglu, Audrey Bowen, Matthew Checketts, Claire Mitchell

**Affiliations:** ^1^ Division of Psychology, Communication and Human Neuroscience, Faculty of Biology, Medicine and Health The University of Manchester Manchester UK; ^2^ Division of Psychology and Mental Health, Faculty of Biology, Medicine and Health The University of Manchester Manchester UK; ^3^ School of Psychology & Neuroscience University of Glasgow Glasgow UK; ^4^ Geoffrey Jefferson Brain Research Centre, The Manchester Academic Health Science Centre Northern Care Alliance & University of Manchester Manchester UK

**Keywords:** facial palsy, stroke, multidisciplinary management, assessment, treatment

## Abstract

**Background:**

Post‐stroke facial palsy significantly impacts patients' communication, eating and overall quality of life. Despite its prevalence, standardised management guidelines are lacking and evidence for assessment and treatment approaches remains limited.

**Aim:**

To describe UK clinical practice for assessing and treating post‐stroke facial palsy and which healthcare professionals play a role.

**Methods:**

A cross‐sectional online survey was conducted using Qualtrics to collect data on participants' professional lives, assessment and treatment practices. UK healthcare professionals in stroke or facial palsy care were recruited through social media, conferences and professional networks.

**Results:**

Ninety‐six out of 150 professionals completed the survey, mainly speech and language therapists (40%) and physiotherapists (43%), including 17 others (occupational therapists, nurses, doctors and orthoptists). Speech and language therapists and physiotherapists were the most likely professions involved in the collaborative management of post‐stroke facial palsy. The most common assessment was clinical observation (84%). Some assessments were more favoured by certain professions, for example, 71% of nurses and doctors used the National Institutes of Health Stroke Scale; speech and language therapists and physiotherapists used the Sunnybrook Facial Grading System and clinical observational methods more than the ‘Other group’. The most commonly used treatments were orofacial exercises (60%) and facial massage (52%).

**Conclusion:**

The study describes current UK practice for managing post‐stroke facial palsy and underscores the critical roles of speech and language therapists and physiotherapists. Now that we know what is used and by whom, we should explore the evidence underpinning this practice to guide assessment and treatment and improve outcomes for stroke survivors.

**WHAT THIS PAPER ADDS:**

*What is already known on this subject*

- Post‐stroke facial palsy affects 45%–60% of acute stroke patients, significantly impacting physical appearance, communication and quality of life. There are currently no clinical guidelines for managing post‐stroke facial palsy, and it is notably absent from national stroke guidelines. Previous international research indicates uncertainty about which professionals should lead facial palsy management after stroke.

*What this paper adds to existing knowledge*

- This is the first UK‐specific survey examining how post‐stroke facial palsy is managed across multiple healthcare professions. Speech and language therapists and physiotherapists are identified as the primary providers of post‐stroke facial palsy care in the UK. The study reveals significant variations in assessment methods, with most clinicians relying on informal observational approaches rather than standardised tools. Common barriers to implementation include a lack of resources, confidence and clear clinical guidelines.

*What are the potential or actual clinical implications of this work?*

- The findings highlight a need for standardised assessment tools and evidence‐based treatment guidelines for post‐stroke facial palsy. Healthcare professionals require specific training in facial palsy assessment and management. Future service development should focus on clarifying professional roles and promoting interdisciplinary collaboration in facial palsy care.

## 1 Introduction

Facial palsy commonly follows stroke (Schimmel et al. 2017; Vaughan et al. 2022; Fabricius et al. 2021), affecting physical appearance, communication, speech, eating, drinking, swallowing and emotional expression (Konecny et al. 2011; Movérare et al. 2017). Effective management is critical for stroke survivors’ well‐being and quality of life (Schimmel et al. 2017). Studies report that 45%–60% of acute stroke patients experience facial palsy (Cattaneo and Pavesi 2014; Mitchell et al. 2021; Volk et al. 2019). To date, there are no published large‐scale studies reporting the prevalence or recovery rates of facial palsy at 6 months post‐stroke. Facial palsy occurs in both ischaemic and haemorrhagic strokes, but robust comparisons of its prevalence and severity between subtypes are challenging due to the lower number of haemorrhagic cases in most studies (Volk et al. 2019; Wongwandee and Hongdusit 2025).

Assessment and treatment of facial palsy after stroke (also known as central facial palsy) often use clinical approaches for people with peripheral facial palsy (Konecny et al. 2011). Common peripheral facial palsy treatments include facial muscle strengthening exercises, biofeedback, facial taping, electrical stimulation, neuromuscular retraining, massage, and, when needed, speech and swallowing exercises (Khan et al. 2022; Karp et al. 2019; van Landingham et al. 2018). Peripheral facial palsy most often results from inflammation of the facial nerve, as seen in Bell's palsy, but can also be caused by infection, trauma, tumours or iatrogenic injury (Nakano et al. 2024; Javed and Daly 2023). It typically causes weakness across the entire side of the face, sometimes with pain, taste changes, synkinesis or reduced tear production (Javed and Daly 2023). In contrast, central facial palsy primarily affects the contralateral lower face due to upper motor neuron lesions, typically sparing the forehead, and may present with other stroke‐related neurological symptoms (Volk et al. 2019; Emos and Agarwal 2023).

Recent studies have increasingly questioned traditional clinical assumptions about central facial palsy. For instance, Wongwandee and Hongdusit (Wongwandee and Hongdusit 2025) reported that upper facial weakness is commonly observed in central facial palsy following acute ischemic stroke, a finding that contradicts classical neurological teaching. Their study underscores the necessity for more sensitive and standardised assessment tools.

Recent research has begun to address the significant gaps in the management of post‐stroke facial palsy, which remains absent from clinical stroke guidelines (Intercollegiate Stroke Working Party 2023; European Stroke Organisation 2022; Heran et al. 2024; Greenberg et al. 2022 Stroke Foundation 2023). For example, Wang et al. (2025) found that acupuncture may offer benefits over conventional therapies for certain outcomes in central facial palsy, though further high‐quality trials are needed. In addition, Shagdar et al. (2025) reviewed advances in computer vision and artificial intelligence for the automated assessment of stroke and facial palsy, highlighting both the promise of these technologies and ongoing challenges such as data availability and model robustness. Despite the involvement of many healthcare professionals in stroke rehabilitation (Intercollegiate Stroke Working Party 2023; Wang et al. 2016), there is still limited research on how post‐stroke facial palsy should be managed and whether assessment and treatment approaches for peripheral facial palsy should be used in stroke (Vaughan et al. 2020). Furthermore, as highlighted by an international survey and systematic review by Vaughan et al. (2022) and a prevalence study by Mitchell et al. (2021), there is no clear information about which professionals should lead or be involved in the management of facial palsy after stroke or what assessments and treatments should be used.

Given the significant impact of facial palsy on stroke survivors, research is needed to understand the multidisciplinary management approaches currently used in clinical practice and guide future research to improve outcomes. This novel study is the first to map the UK landscape of post‐stroke facial palsy care, providing crucial insights into current clinical practice. The aim of this study is to understand which UK healthcare professionals are involved in the diagnosis and treatment of post‐stroke facial palsy, and to identify the specific assessments and interventions used by different occupational groups.

## 2 Method

This study was a cross‐sectional, online survey collecting self‐reported data from healthcare professionals involved in stroke facial palsy care in the UK and received research ethics approval from the University of Manchester, 2023‐16299‐29326. Reporting follows the Checklist for Reporting of Survey Studies (CROSS) (Sharma et al. 2021).

A convenience and voluntary response sampling approach was used to gather responses from healthcare professionals working in stroke and facial palsy care across the UK. Twelve different health professional networks, including stroke and facial palsy networks (see Acknowledgements for the list of networks), were contacted via email with an invitation to participate in the survey, which was hosted on Qualtrics. To increase participation, the principal investigator sent reminder emails approximately 1 month after the initial invitation. Additionally, the survey was promoted through six social media advertisements on X/Twitter and LinkedIn, as well as conference announcements (UK Stroke Forum), to increase awareness. Participants were encouraged to complete the survey voluntarily, and no specific geographical clusters or institutions were targeted. The survey remained open from July 2023 until December 2023.

Survey content and layout were collaboratively developed in four stages:

- Stage 1: The first author conducted a literature review, extracting systematic reviews on post‐stroke facial palsy and reported methods for the assessment and treatment of facial palsy after stroke were extracted and presented to other authors.
- Stage 2: The first author created the first version of the survey. Consensus was reached amongst the authors on suggested changes, and an online survey was then developed.
- Stage 3: This online version was piloted by the authors and colleagues in terms of functionality, ease of completion and data format: three times with the authors and once with colleagues (two speech and language therapists, two physiotherapists, one psychologist, two biostatisticians and one occupational therapist), representing a sample with a professional background similar to the study's target population.
- Stage 4: A revised online survey was distributed to reach health professionals.

The survey was designed on Qualtrics and intended to take around 15 min to complete (Supporting Information—Survey Questions). Participants only completed one round. The survey did not collect identifying information such as name, date of birth or electronic information such as IP address. While IP addresses were not stored, Qualtrics blocked multiple submissions from the same IP address, further reducing the chance of repeated participation. There were three parts. Part 1 included 10 background questions on participants’ professional training, work setting and clinical experience. Part 2 included 31 questions, focused on assessment methods for facial palsy (Table 1). Participants were asked about 14 standardised measurement tools and their reasons for using them with closed and open questions about other assessment approaches. Part 3 included 16 questions and asked about six treatment methods (Table 2) the participants used or were aware of, and the reasons for their use via closed and open questions. Reasons for not using treatments were also asked.

**TABLE 1:** Assessments specified in the survey.

Name of assessment method	Type	Brief description
Clinical observation	Observational/Clinician‐reported	Visual inspection of facial symmetry and movement; subjective
House‐Brackmann Facial Grading Scale (HBGS) (House and Brackmann 1985)	Clinician‐reported	6‐point scale providing an overall grade of facial nerve function
Sunnybrook Facial Grading System (SFGS) (Ross et al. 1996)	Clinician‐reported	Rates symmetry, synkinesis and movement, providing more detailed info
Facial Disability Index (FDI) (VanSwearingen and Brach 1996)	Patient‐reported	Questionnaire assessing physical and social function
Facial Clinimetric Evaluation (FaCE) (Kahn et al. 2001)	Patient‐reported	Questionnaire on facial function and psychological aspects of facial palsy
Electronic Facial Paralysis Assessment (eFACE) (Banks and al. 2015)	Clinician‐reported	Digital clinician‐graded facial function scale
Stennert Index (Stennert et al. 1977)	Clinician‐reported	Rates facial function and synkinesis
Photographic analysis (Svensson et al. 1992)	Clinician‐reported	Use of photographs to assess facial symmetry and movement
Video analysis (Konecny et al. 2011, Schimmel et al. 2011)	Clinician‐reported	Video recording and analysis of facial movement
National Institutes of Health Stroke Scale (NIHSS) (Brott et al. 1989)	Clinician‐reported	Stroke severity scale; includes facial palsy item
Face Arm Speech Test (FAST) (Harbison et al. 1999)	Clinician‐reported	Rapid stroke screening tool; includes facial droop
Recognition of Stroke in the Emergency Room (ROSIER) (Nor et al. 2005)	Clinician‐reported	Stroke recognition tool; includes facial weakness
Cincinnati Prehospital Stroke Scale (CPSS) (Kothari and al. 1999)	Clinician‐reported	Prehospital stroke scale; includes facial droop
Los Angeles Prehospital Stroke Scale (LAPSS) (Kidwell et al. 2000)	Clinician‐reported	Prehospital stroke scale; includes facial paralysis

**TABLE 2:** Treatments specified in the survey.

Name of treatment method	Brief description
Orofacial exercises (Pereira et al. 2011)	Exercises targeting facial muscles to improve strength, coordination and movement
Facial massage (Choi and Shin 2016)	Manual manipulation of facial tissues to improve circulation and reduce stiffness (individual or therapist)
Electrical stimulation (Burelo‐Peregrino et al. 2020)	Use of electrical currents to stimulate facial muscles
EMG‐Biofeedback (Svensson et al. 1992)	Use of electromyography to provide feedback on muscle activity during exercises
Proprioceptive neuromuscular facilitation (Silva et al. 2022)	Techniques to enhance neuromuscular control and coordination of facial muscles
Heat therapy (Shafshak 2006)	Application of heat to facial tissues to promote relaxation and circulation

**FIGURE 1:**
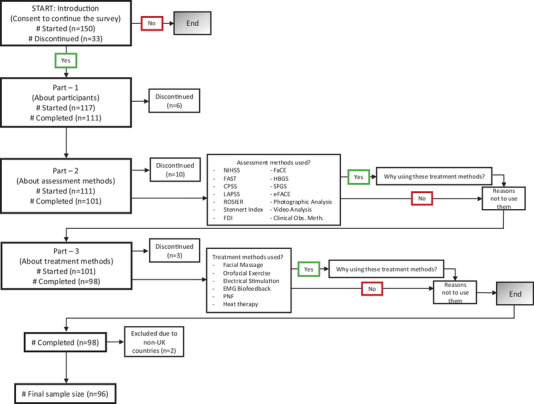
Recruitment flowchart.

Descriptive and frequency statistics were used to analyse the participants' characteristics and relationships with the assessment and treatment methods. Frequency and Likert scale data were tallied, tabulated and graphically displayed. Chi‐squared tests of independence were conducted to examine whether there was a relationship between participants' occupation and their use of assessments and treatments. If the *p* value was less than 0.05, post‐hoc analyses were performed to identify which occupation groups used specific assessment and treatment types more or less frequently. Bonferroni correction was applied to the post‐hoc analysis to adjust for multiple comparisons. All the quantitative analyses were performed in IBM SPSS Version 29. Statistical significance was concluded using the threshold of *p* ≤ 0.05. No weighting of items or propensity scores was used in case the sample was non‐representative, and no sensitivity analysis was conducted. The participant flow and decision points of the survey are illustrated in Figure 1.

## 3 Results

The raw, fully anonymous data were downloaded in an Excel spreadsheet from the Qualtrics platform in January 2024. Only completed surveys from UK‐based participants were included in the final analysis. A final sample size of 96 participants was obtained from 150 surveys initiated, and 111/150 who provided data about themselves in Part 1. Figure 1 shows how many people completed and discontinued the parts of the survey.

### 3.1 Participant Demographics and Interdisciplinary Collaboration

Table 3 shows the numbers of respondents according to their professions, geographical locations, clinical practice areas and length of experience in their professions. Most participants were physiotherapists (41/96, 43%), followed by speech and language therapists (38/96, 40%). A smaller proportion of doctors, nurses, occupational therapists and orthoptists grouped as ‘other’ reported being involved in the management of post‐stroke facial palsy (*n* = 17) compared to physiotherapists and speech and language therapists.

**TABLE 3:** Characteristics of participants.

	SLT	PT	Other				Total
			OT	Nurse	Doctor	Orthoptist	
Demographics	(*n* = 38)	(*n* = 41)	(*n* = 2)	(*n* = 9)	(*n* = 5)	(*n* = 1)	(*n* = 96)
Country, *n (%)*							
England	35 (92)	39 (95)	2 (100)	7 (78)	5 (100)	1 (100)	89 (93)
Scotland and Wales	3 (8)	2 (5)	0 (0)	2 (22)	0 (0)	0 (0)	7 (7)
Work setting, *n (%)*							
Inpatient: Acute and rehab stroke	28 (62)	23 (50)	2 (67)	7 (64)	5 (100)	1 (50)	66 (59)
Community: Discharge and long‐term support	15 (33)	18 (39)	1 (33)	3 (27)	0 (0)	0 (0)	37 (33)
Other	2 (4)	5 (11)	0 (0)	1 (9)	0 (0)	1 (50)	9 (8)
Experience in clinical practice, *n (%)*							
0–5 years	16 (42)	2 (5)	0 (0)	2 (22)	1 (20)	0 (0)	21 (22)
5–10 years	11 (29)	5 (12)	1 (50)	3 (33)	0 (0)	0 (0)	20 (21)
Over 10 years	11 (29)	34 (83)	1 (50)	4 (44)	4 (80)	1 (100)	55 (57)

*Note*: For the work settings, respondents were allowed to select more than one response.

Abbreviations: OT, occupational therapist; PT, physiotherapist; SLT, speech and language therapist.

The majority of participants were based in England. Nearly 60% worked in Acute Stroke and Stroke Rehabilitation Inpatient and had more than ten years of clinical experience in their profession.

The survey respondents were most likely to select speech and language therapists (79%, *n* = 46/58) and physiotherapists (49%, *n* = 27/55) as the healthcare professionals they work with in managing post‐stroke facial palsy. When analysing these collaborations, we only included responses from individuals belonging to a different profession. For example, when reporting collaboration with speech and language therapists, we excluded responses from speech and language therapist participants. (Table S1).

### 3.2 Assessment Methods Used in Clinical Practice

**FIGURE 2:**
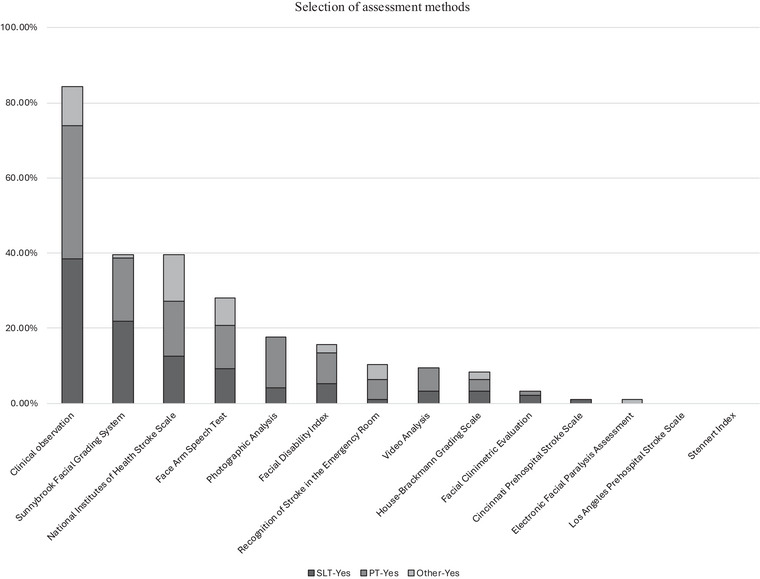
Assessments used in the management of post‐stroke facial palsy by professional group.

Of the 14 assessment methods provided, participants reported that two, the Los Angeles Prehospital Stroke Scale and the Stennert Index, were not used (Figure 2). The most frequently used assessment across all professional groups was clinical observation, defined here as subjectively examining the patient's face for asymmetry or differences between the two sides (e.g., movement or expression). Clinical observation was selected by 84% (*n* = 81/96) of healthcare professionals, with speech and language therapists (97%, *n* = 37/38) most likely to use it compared to all other occupational groups (*χ* ^2^ = 13.3, df = 2, *p* = 0.001, Cramer's V = 0.37, on post‐hoc analysis with Bonferroni correction *p* < 0.0167) (Table S2).

Sunnybrook facial grading system and the National Institutes of Health Stroke Scale were the joint second most commonly used assessment (39%, *n* = 38/96). Speech and language therapists (55%, *n* = 21/38) and physiotherapists (39%, *n* = 16/41) were significantly more likely to use Sunnybrook facial grading system than the ‘Other’ group (6%, *n* = 1/17), whereas the ‘Other’ group (71%, *n* = 12/17) were more likely to use National Institutes of Health Stroke Scale than speech and language therapists (32%, *n* = 12/38) and physiotherapists (34%, *n* = 14/41) (Table S2 and Figure S1).

The only other assessment that had a relationship with a certain profession was photographic analysis (*χ* ^2^ = 10.517, df = 2, *p* = 0.005), and only for physiotherapists (32%, *n* = 13/41) who used it more frequently than the ‘Other’ group (0%, *n* = 0/17) (Table S2).

The most common reason for using clinical observational methods was professional choice, while the most common reason for using the National Institutes of Health Stroke Scale was department policy (Table S3).

Only five participants stated that they used assessments that were not suggested in the survey. These were: Clinical Outcomes in Routine Evaluation‐10 and synkinesis assessment tools (*n* = 1), photographic documentation (*n* = 1), informal cranial nerve assessment (*n* = 1), bedside oromotor assessment as part of swallow assessment (*n* = 1) and facial palsy case history (*n* = 1).

### 3.3 Treatment Approaches Used in Clinical Practice

**FIGURE 3:**
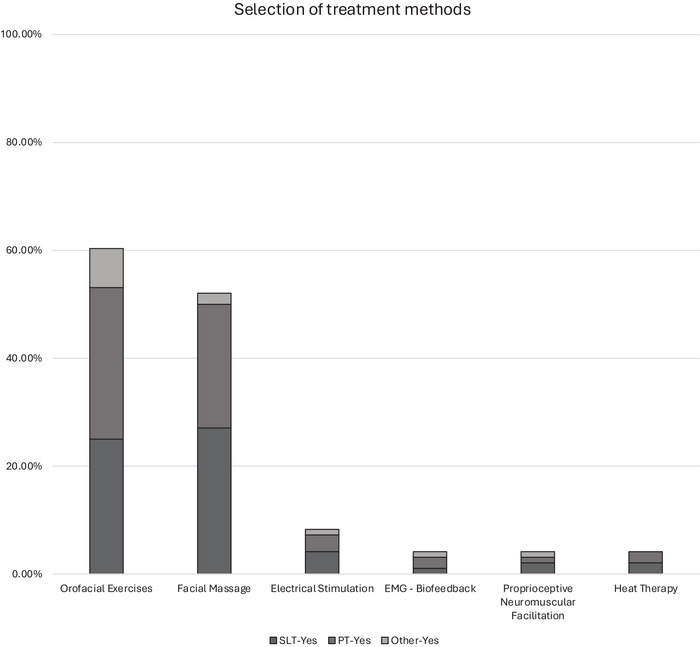
Methods used in the management of post‐stroke facial palsy.

The most commonly used treatment methods for post‐stroke facial palsy are orofacial exercises and facial massage, used by 60% and 52% of professionals, respectively (Figure 3). Other treatments were rarely used by anyone (4%–8%). Speech and language therapists and physiotherapists were the primary providers of interventions, and very few other healthcare professionals offered any of these treatments (Table S4 and Figure S2). The most common reason for using orofacial exercises and facial massage was professional choice (Table S5).

Eighteen participants (speech and language therapists and physiotherapists only) suggested additional treatments not included in the survey. These were: facial taping (*n* = 14), stretches (*n* = 4), mirror therapy (*n* = 1), advice on eating, drinking, speaking modifications (*n* = 1), facial neuromuscular retraining (*n* = 1) and emotional/psychological support (*n* = 1). Specifically, 14 out of the 18 respondents who answered ‘Yes’ to this open question mentioned using facial taping, either alone or in combination with other approaches such as stretches, mirror therapy or neuromuscular retraining.

### 3.4 Education and Training of Healthcare Professionals

When examining why healthcare professionals did not choose to use some of the management approaches, the three main reasons generally cited were lack of resources, confidence and clinical guidelines (Table S6).

Regarding training for post‐stroke facial palsy, both in assessment and treatment, respondents most commonly reported receiving training through professional training (34% for assessment, 32% for treatment), an undergraduate degree (30% for both) or other sources, such as in‐service training and peer training (24% for assessment, 27% for treatment). Smaller proportions cited postgraduate education (7% for both assessment and treatment). Notably, about a quarter of participants stated that they had not received any specific training in either the assessment or management of post‐stroke facial palsy disorders (Table S1).

## 4 Discussion

This novel study provides the first examination of post‐stroke facial palsy care across UK healthcare professions and reveals that speech and language therapists and physiotherapists are the primary providers involved in the care of post‐stroke facial palsy in the UK. Our findings indicate significant variations in assessment methods, with a predominant reliance on informal observational approaches rather than standardised tools. Common treatment strategies include orofacial exercises and facial massage, but healthcare professionals feel that a lack of resources, confidence and clear clinical guidelines hinders implementation. Additionally, the survey shows varying degrees of interaction between different professional groups, but further work would be needed to determine the impact of collaborative working on patient care and clarify the distribution of responsibilities amongst the specialities involved.

The finding that speech and language therapists and physiotherapists are the professional groups most involved with post‐stroke facial palsy rehabilitation aligns with their expertise in facial muscles (Vaughan et al. 2020; Enderby et al., 2013; Therapists 2014; Konecny et al. 2014; Tawiah et al. 2021). However, Vaughan et al. (2022) reported role ambiguity between these professions in addressing post‐stroke facial palsy in their international survey study, possibly due to unclear clinical guidelines. Notably, Vaughan et al. (2022) had a predominantly speech and language therapist sample, while our study included a more balanced mix of SLTs and PTs and other professionals, offering a broader view of multidisciplinary practice in the UK. While both studies highlight role ambiguity, our results suggest this may be particularly pronounced in the UK due to the lack of national guidelines and challenges related to resource allocation and service provision. This uncertainty could impact treatment and research directions, with the professions unclear who should lead or even whose responsibility it is. Other research highlights the need for interdisciplinary collaboration and future guidance must consider how professions should work together (Clarke and Forster 2015; Butler and Grobbelaar 2017).

### 4.1 Implications of Assessment Practices

We found that speech and language therapists and physiotherapists were most likely to use informal clinical observations and the Sunnybrook scale, which aligns with an international survey of mostly speech and language therapists (Vaughan et al. 2022). Although both professions use similar assessment tools, our UK‐based survey included a broader range of healthcare professionals and revealed a wider perspective of professions, especially speech and language therapists and physiotherapists, a detail not highlighted in Vaughan et al. (2022). The involvement of both professions could offer a more comprehensive view of facial function evaluation.

The preference for informal methods, while potentially efficient in resource‐constrained settings, raises concerns about consistency, reliability and the ability to accurately diagnose and track patient progress effectively across different settings and studies (Coelho et al. 2005). This underscores the need for further research, evaluation and standardisation of existing tools.

We found in clinical practices the Sunnybrook facial grading system (40%) was preferred over the House‐Brackmann Facial Grading System (8%), although published reviews of assessment for post‐stroke facial palsy often use the House‐Brackmann Facial Grading System as a quantitative outcome measure (Fabricius et al. 2021; Vaughan et al. 2020). This clinical preference may stem from recent evidence supporting Sunnybrook's reliability on post‐stroke facial palsy (Tramontano et al. 2021). However, it is important to note that this research is still in its early stages with small sample sizes. As seen elsewhere, professional roles also influenced assessment tool choice (Vaughan et al. 2022). While nurses and doctors appeared to favour the National Institutes of Health Stroke Scale, perhaps because it is routinely used for initial stroke screening and reported in the UK national audit (Sentinel Stroke National Audit Programme 2019), speech and language therapists and physiotherapists use the more detailed Sunnybrook facial grading system. In addition, assessment tool selection varies based on healthcare professionals' training needs, potentially indicating an evolving clinical practice in post‐stroke facial palsy evaluation.

### 4.2 Evaluation of Treatment Approaches

Our finding that orofacial exercises and facial massage were the most commonly used treatment methods for post‐stroke facial palsy aligns with Vaughan et al. (2022). Orofacial exercises and facial massage potentially target facial muscles to improve function and appearance (Vaughan et al. 2022; Konecny et al. 2014). However, our research and previous studies (Vaughan et al. 2022; Fabricius et al. 2021; Vaughan et al. 2020) identified significant barriers to implementing these treatments. These include a lack of resources, confidence and clear evidence‐based guidelines. While various interventions exist, including active muscle strengthening and passive massage techniques (Fabricius et al. 2021; Vaughan et al. 2020), high‐quality evidence supporting their efficacy in stroke patients is lacking. Notably, in the management of peripheral facial palsy, recent expert consensus (Neville et al. 2024) discourages non‐specific treatments such as gross facial exercises and electrical stimulation. Similarly, a Cochrane review of peripheral facial palsy found only low‐quality evidence supporting personalised facial exercises (Teixeira et al. 2011). Despite these, such interventions remain widely used, underscoring the challenge of changing established clinical practices. However, developing evidence‐based guidelines is important and should include robust research, for example, evaluating the most effective treatments for post‐stroke facial palsy. In the meantime, healthcare professionals should continue to provide care according to their local guidelines and consider facilitating research.

Participants suggested several additional treatment approaches beyond those listed in the main survey, including facial taping, stretches, mirror therapy, neuromuscular retraining, advice on speech, communication and oral motor function and psychological support. The evidence base for these interventions in post‐stroke facial palsy is variable. For example, mirror therapy has been explored in preliminary studies for post‐stroke facial palsy, with some suggesting potential benefits (Kang et al. 2017). Interventions targeting speech, communication and oral motor function, as well as psychological support, are recognised as important aspects of holistic care in stroke rehabilitation (Schimmel et al. 2017; Baker et al. 2022). However, for other approaches such as facial taping, stretches and neuromuscular retraining, there is currently limited or insufficient evidence specific to post‐stroke facial palsy and further research is needed to establish their effectiveness in this population.

The diversity of additional treatments suggested highlights the evolving clinical practice and the need for further research into their effectiveness. In addition, the face is controlled by a complex network involving motor, sensory and limbic (emotional) pathways (Ottaiano et al. 2023), making rehabilitation particularly challenging. Facial function is essential not only for movement and expression, but also for sensation and emotional communication (Krumhuber et al. 2023). As such, therapeutic intervention for facial palsy requires a holistic approach; simple exercise‐based protocols may not fully address the multifaceted nature of facial impairment after stroke. Our findings underscore the need for further research specifically targeting interventions for post‐stroke cohorts.

### 4.3 Education and Training

Our study revealed considerable variability in the training backgrounds of UK‐based speech and language therapists and physiotherapists regarding post‐stroke facial palsy. While a notable proportion of both professions reported receiving some training at the undergraduate level, a significant number indicated having no formal training in this area, with approximately a quarter of participants lacking specific education on assessment and treatment. This gap may reflect the absence of central facial palsy content in many UK undergraduate curricula for physiotherapy and speech and language therapy programs, as well as the lack of national clinical guidelines or standardised training pathways for this condition. These findings underscore the need for dedicated education and training programs, as well as robust continuing professional development and clear clinical guidelines, to ensure consistent, high‐quality care for individuals with post‐stroke facial palsy.

Consensus guidelines and evidence‐based approaches for peripheral facial palsy could provide a valuable model for developing best practices in post‐stroke facial palsy. Recent expert consensus (Neville et al. 2024) emphasises the importance of standardised assessment tools, patient education, individualised therapy and home programs, while discouraging non‐specific treatments like gross facial exercises and electrical stimulation. Adapting these structured, multidisciplinary and patient‐centred principles from peripheral facial palsy management (Khan et al. 2022; Karp et al. 2019; van Landingham et al. 2018; Neville et al. 2024) could help guide the development of effective, evidence‐based guidelines for post‐stroke facial palsy, where such frameworks are currently lacking.

### 4.4 Strengths, Limitations and Future Directions

This study's strengths include its insights into current practices across speech and language therapists and physiotherapists involved in post‐stroke facial palsy management. Its limitations include potential self‐selection bias, limited generalisability due to the overrepresentation of certain regions, recall bias from self‐reported data and the lack of patient perspectives. Another limitation is that the survey did not differentiate between non‐speech oral motor exercises and speech‐related oral tasks; thus, we could not determine the specific type of oral motor exercises utilised by professionals. Understanding the current clinical management context in the UK gives useful indications of how to build the evidence base and directions for future work, with the longer‐term intention of contributing to future clinical guidelines for post‐stroke facial palsy.

## 5 Conclusion

This study highlights the significant involvement of both speech and language therapists and physiotherapists in managing post‐stroke facial palsy, but also reveals variability in assessment and treatment approaches. Our findings reinforce the urgent need for the development of evidence‐based, interdisciplinary clinical practice guidelines to support standardisation of care. Future efforts should prioritise systematic reviews to evaluate the effectiveness, feasibility and acceptability of current assessment and treatment methods, as well as the implementation of longitudinal studies to better understand the progression and long‐term impact of facial palsy after stroke. Importantly, to ensure that future guidelines are robust and relevant, the development of a core outcome set, agreed upon by clinicians, researchers and patient representatives, would be an important step in harmonising outcome measurement across studies and clinical practice. Additionally, collaborative initiatives involving clinicians, researchers and patient representatives will be essential to ensure that emerging guidelines are both practical and patient‐centred. Such steps are crucial to improving the quality and consistency of care for individuals with post‐stroke facial palsy.

## Ethics Statement

Ethics approval was granted by the University of Manchester, 2023‐16299‐29326.

## Conflicts of Interest

The author(s) declared no potential conflicts of interest with respect to the research, authorship, and/or publication of this article.

## Supporting information




**Supplementary Table‐1**: Detailed characteristics of demographics
**Supplementary Table‐2**: Chi‐Squared test for assessments
**Supplementary Table‐3**: Reasons to use assessments
**Supplementary Table‐4**: Chi‐Squared test for treatments
**Supplementary Table‐5**: Reasons to use treatments
**Supplementary Table 6**: Reasons for not using the treatments

**Supporting**: jlcd70127‐sup‐0002‐FigureS1.png

**Supporting**: jlcd70127‐sup‐0003‐FigureS2.png

**Supporting**: jlcd70127‐sup‐0004‐Survey‐Questions.docx

**Supporting**: jlcd70127‐sup‐0005‐CROSS.docx

## Data Availability

All data produced in the present study are available upon reasonable request to the authors.
