# Supplementary material for: Managing Facial Palsy After Stroke: Results From an Online Survey of Health Professionals
Source: Int J Lang Commun Disord. 2025 Sep 16;60(5):e70127. doi: 10.1111/1460-6984.70127 (PMC12439456; doi:10.1111/1460-6984.70127)
Supplement: Supplementary file 1 — Supplementary Table‐1: Detailed characteristics of demographics Supplementary Table‐2: Chi‐Squared test for assessments Supplementary Table‐3: Reasons to use assessments Supplementary Table‐4: Chi‐Squared test for treatments Supplementary Table‐5: Reasons to use treatments Supplementary Table 6: Reasons for not using the treatments [file JLCD-60-0-s001.docx]

Supplementary Table-1

|  | SLT | PT | Other | | | | Total |
| --- | --- | --- | --- | --- | --- | --- | --- |
|  |  |  | OT | Nurse | Doctor | Orthoptist |  |
|  | (n=38) | (n=41) | (n=2) | (n=9) | (n=5) | (n=1) | (n=96) |
| Sources of referrals for post-stroke facial palsy, *n (%)* | | | | | | | |
| Referred specifically | 12 (32) | 5 (12) | 0 (0) | 2 (22) | 1 (20) | 0 (0) | 20 (21) |
| Picked up on the assessment | 25 (66) | 35 (85) | 2 (100) | 6 (67) | 4 (80) | 1 (100) | 73 (76) |
| Both of them | 1 (27) | 1 (2) | 0 (0) | 1 (11) | 0 (0) | 0 (0) | 3 (3) |
| Training received on assessments for post-stroke facial palsy, *n (%)* | | | | | | | |
| Undergraduate degree | 13 (34) | 10 (24) | 0 (0) | 1 (11) | 4 (80) | 1 (100) | 29 (30) |
| Post-graduate degree | 7 (18) | 0 (0) | 0 (0) | 1 (11) | 2 (40) | 0 (0) | 10 (10) |
| Professional training | 9 (24) | 15 (37) | 0 (0) | 6 (67) | 2 (40) | 1 (100) | 33 (34) |
| Other | 11 (29) | 9 (22) | 1 (50) | 2 (22) | 0 (0) | 0 (0) | 23 (24) |
| None | 5 (13) | 15 (37) | 1 (50) | 1 (11) | 0 (0) | 0 (0) | 24 (25) |
| Training received on treatments for post-stroke facial palsy, *n (%)* | | | | | | | |
| Undergraduate degree | 14 (37) | 10 (24) | 0 (0) | 1 (11) | 3 (60) | 1 (100) | 29 (30) |
| Post-graduate degree | 4 (11) | 0 (0) | 0 (0) | 0 (0) | 2 (40) | 0 (0) | 6 (6) |
| Professional training | 7 (18) | 16 (39) | 0 (0) | 6 (67) | 1 (20) | 1 (100) | 31 (32) |
| Other | 14 (37) | 11 (27) | 1 (50) | 0 (0) | 0 (0) | 0 (0) | 26 (27) |
| None | 5 (13) | 14 (34) | 1 (50) | 3 (33) | 1 (20) | 0 (0) | 24 (25) |
| Professions work together to manage post-stroke facial palsy, *n (%)* | | | | | | | |
| SLT | ~~-~~ | 34 (83) | 2 (100) | 7 (78) | 3 (60) | 0 (0) | 46 (79) |
| PT | 17 (45) | ~~-~~ | 0 (0) | 6 (67) | 4 (80) | 0 (0) | 27 (49) |
| OT | 3 (5) | 8 (20) | ~~-~~ | 5 (56) | 2 (40) | 0 (0) | 18 (19) |
| Nurse | 4 (7) | 9 (22) | 1 (50) | ~~-~~ | 1 (20) | 0 (0) | 15 (17) |
| Doctor | 5 (8) | 6 (15) | 1 (50) | 5 (56) | - | 0 (0) | 17 (19) |
| Psychologist | 2 (3) | 4 (10) | 0 (0) | 1 (11) | 1 (20) | 0 (0) | 8 (8) |
| Other | 4 (7) | 2 (5) | 0 (0) | 1 (11) | 0 (0) | 1 (100) | 8 (8) |

For training received on assessment and treatment methods for post-stroke facial palsy and professions work together to manage post-stroke facial palsy, respondents were allowed to select more than one response. SLT: speech and language therapist; PT: physiotherapist; OT: occupational therapist.

Supplementary Table-2 Chi-Squared test for assessments

| Chi Squared Test | | Yes | No | χ² | df | p | Cramer's V | Post-hoc Comparisons  (p-values) |
| --- | --- | --- | --- | --- | --- | --- | --- | --- |
| NIHSS | SLT (n=38) | 12 (32%) | 26 (68%) | 8.35 | 2 | 0.015* | 0.295 | SLT vs PT: 0.808 SLT vs Other: 0.007** PT vs Other: 0.011** |
|  | PT (n=41) | 14 (34%) | 27 (66%) |  |  |  |  |  |
|  | Other (n=17) | 12 (71%) | 5 (29%) |  |  |  |  |  |
| FAST | SLT (n=38) | 9 (24%) | 29 (76%) | 1.83 | 2 | 0.399 | 0.138 |  |
|  | PT (n=41) | 11 (27%) | 30 (73%) |  |  |  |  |  |
|  | Other (n=17) | 7 (41%) | 10 (59%) |  |  |  |  |  |
| CPSS | SLT (n=38) | 1 (3%) | 37 (97%) | 1.54 | 2 | 0.462 | 0.127 |  |
|  | PT (n=41) | 0 (0%) | 41 (100%) |  |  |  |  |  |
|  | Other (n=17) | 0 (0%) | 17 (100%) |  |  |  |  |  |
| ROSIER | SLT (n=38) | 1 (3%) | 37 (97%) | 5.73 | 2 | 0.057 | 0.245 |  |
|  | PT (n=41) | 5 (12%) | 36 (88%) |  |  |  |  |  |
|  | Other (n=17) | 4 (24%) | 13 (77%) |  |  |  |  |  |
| FDI | SLT (n=38) | 5 (13%) | 33 (87%) | 0.83 | 2 | 0.658 | 0.093 |  |
|  | PT (n=41) | 8 (20%) | 33 (81%) |  |  |  |  |  |
|  | Other (n=17) | 2 (12%) | 15 (88%) |  |  |  |  |  |
| FaCE | SLT (n=38) | 2 (5%) | 36 (95%) | 1.18 | 2 | 0.553 | 0.111 |  |
|  | PT (n=41) | 1 (2%) | 40 (98%) |  |  |  |  |  |
|  | Other (n=17) | 0 (0%) | 17 (100%) |  |  |  |  |  |
| HBGS | SLT (n=38) | 3 (8%) | 35 (92%) | 0.32 | 2 | 0.849 | 0.058 |  |
|  | PT (n=41) | 3 (7%) | 38 (93%) |  |  |  |  |  |
|  | Other (n=17) | 2 (12%) | 15 (92%) |  |  |  |  |  |
| SFGS | SLT (n=38) | 21 (55%) | 17 (45%) | 11.98 | 2 | 0.002* | 0.353 | SLT vs PT: 0.148 SLT vs Other: <0.001** PT vs Other: 0.012** |
|  | PT (n=41) | 16 (39%) | 25 (61%) |  |  |  |  |  |
|  | Other (n=17) | 1 (6%) | 16 (94%) |  |  |  |  |  |
| eFACE | SLT (n=38) | 0 (0%) | 38 (100%) | 4.69 | 2 | 0.096 | 0.221 |  |
|  | PT (n=41) | 0 (0%) | 41 (100%) |  |  |  |  |  |
|  | Other (n=17) | 1 (6%) | 16 (94%) |  |  |  |  |  |
| Photographic Analysis | SLT (n=38) | 4 (11%) | 34 (90%) | 10.517 | 2 | 0.005* | 0.331 | SLT vs PT: 0.022 SLT vs Other: 0.299 PT vs Other: 0.006** |
|  | PT (n=41) | 13 (32%) | 28 (68%) |  |  |  |  |  |
|  | Other (n=17) | 0 (0.0%) | 17 (100%) |  |  |  |  |  |
| Video Analysis | SLT (n=38) | 3 (8%) | 35 (92%) | 3.191 | 2 | 0.203 | 0.182 |  |
|  | PT (n=41) | 6 (15%) | 35 (85%) |  |  |  |  |  |
|  | Other (n=17) | 0 (0%) | 17 (100%) |  |  |  |  |  |
| Clinical Observation Methods | SLT (n=38) | 37 (97%) | 1 (3%) | 13.35 | 2 | 0.001* | 0.373 | SLT vs PT: 0.058 SLT vs Other: <0.001** PT vs Other: 0.089 |
|  | PT (n=41) | 34 (83%) | 7 (17%) |  |  |  |  |  |
|  | Other (n=17) | 10 (59%) | 7 (41%) |  |  |  |  |  |

Post-hoc comparisons were conducted for assessments with significant Chi-Square results. The Bonferroni correction was applied to adjust for multiple comparisons. Significant p-values (p < 0.05) are indicated with *. P-values that remain significant after Bonferroni correction (p < 0.017) are indicated with **. NIHSS: national institutes of health stroke scale; FAST: face, arms, speech, time; CPSS: cincinnati prehospital stroke scale; ROSIER: recognition of stroke in the emergency room; FDI: facial disability index; FaCE: facial clinimetric evaluation; HBGS: house-brackmann grading system; SFGS: sunnybrook facial grading system; eFACE: electronic facial paralysis assessment.

Supplementary Table-3 Reasons to use assessments

|  | Department policy | Professional choice | Useful to indicate facial palsy after stroke | Useful as part of a wider assessment | Recommended by practice guidelines | Widely used by others | Other |
| --- | --- | --- | --- | --- | --- | --- | --- |
| NIHSS | 24 | 5 | 4 | 21 | 18 | 14 | 1 |
| FAST | 6 | 7 | 8 | 18 | 8 | 8 | 1 |
| CPSS | 0 | 1 | 0 | 0 | 1 | 0 | 0 |
| LAPSS | 0 | 0 | 0 | 0 | 0 | 0 | 0 |
| ROSIER | 5 | 2 | 1 | 5 | 1 | 2 | 1 |
| Stennert Index | 0 | 0 | 0 | 0 | 0 | 0 | 0 |
| FDI | 1 | 10 | 3 | 8 | 3 | 10 | 0 |
| FaCE | 1 | 2 | 1 | 3 | 1 | 3 | 0 |
| HBGS | 1 | 5 | 3 | 2 | 1 | 5 | 0 |
| SFGF | 5 | 22 | 22 | 15 | 5 | 26 | 6 |
| eFACE | 0 | 1 | 0 | 0 | 0 | 0 | 0 |
| Photographic analysis | 2 | 14 | 12 | 10 | 2 | 11 | 5 |
| Video analysis | 1 | 6 | 6 | 6 | 1 | 6 | 2 |
| Clinical Observation methods | 5 | 53 | 33 | 47 | 5 | 23 | 8 |

NIHSS: national institutes of health stroke scale; FAST: face, arms, speech, time; CPSS: cincinnati prehospital stroke scale; ROSIER: recognition of stroke in the emergency room; FDI: facial disability index; FaCE: facial clinimetric evaluation; HBGS: house-brackmann grading system; SFGS: sunnybrook facial grading system; eFACE: electronic facial paralysis assessment.

Supplementary Table-4 Chi-Squared test for treatments

| Chi Square Test | |  | Yes | No | χ² | df | p | Cramer's V | Post-hoc Comparisons (p-values) |
| --- | --- | --- | --- | --- | --- | --- | --- | --- | --- |
| Facial Massage | SLT (n=38) |  | 26 (68%) | 12 (32%) | 15.178 | 2 | < 0.001* | 0.398 | SLT vs PT: 0.179 SLT vs Other: <0.001** PT vs Other: 0.003** |
|  | PT (n=41) |  | 22 (54%) | 19 (46%) |  |  |  |  |  |
|  | Other (n=17) |  | 2 (12%) | 15 (88%) |  |  |  |  |  |
| Orofacial Exercise | SLT (n=38) |  | 24 (63%) | 14 (37%) | 3.258 | 2 | 0.196 | 0.184 |  |
|  | PT (n=41) |  | 27 (66%) | 14 (34%) |  |  |  |  |  |
|  | Other (n=17) |  | 7 (41%) | 10 (59%) |  |  |  |  |  |
| EMG - Biofeedback | SLT (n=38) |  | 1 (3%) | 37 (97%) | 0.402 | 2 | 0.818 | 0.065 |  |
|  | PT (n=41) |  | 2 (5%) | 39 (95%) |  |  |  |  |  |
|  | Other (n=17) |  | 1 (6%) | 16 (94%) |  |  |  |  |  |
| Heat therapy | SLT (n=38) |  | 2 (5%) | 36 (95%) | 0.906 | 2 | 0.636 | 0.097 |  |
|  | PT (n=41) |  | 2 (5%) | 39 (95%) |  |  |  |  |  |
|  | Other (n=17) |  | 0 (0%) | 17 (100%) |  |  |  |  |  |
| Electrical Stimulation | SLT (n=38) |  | 4 (11%) | 34 (90%) | 0.428 | 2 | 0.807 | 0.067 |  |
|  | PT (n=41) |  | 3 (7%) | 38 (93%) |  |  |  |  |  |
|  | Other (n=17) |  | 1 (6%) | 16 (94%) |  |  |  |  |  |
| Proprioceptive Neuromuscular Facilitation | SLT (n=38) |  | 2 (5%) | 36 (95%) | 0.546 | 2 | 0.761 | 0.075 |  |
|  | PT (n=41) |  | 1 (2%) | 40 (98%) |  |  |  |  |  |
|  | Other (n=17) |  | 1 (6%) | 16 (94%) |  |  |  |  |  |

Post-hoc comparisons were conducted for treatments with significant Chi-Square results. The Bonferroni correction was applied to adjust for multiple comparisons. Significant p-values (p < 0.05) are indicated with *. P-values that remain significant after Bonferroni correction (p < 0.017) are indicated with **.

Supplementary Table-5 Reasons to use treatments

|  | Department policy | Professional choice | Useful to treat facial palsy after stroke | Useful as a general neurological probe | Recommended by practice guidelines | Widely used by others | Other |
| --- | --- | --- | --- | --- | --- | --- | --- |
| Facial massage | 4 | 38 | 31 | 8 | 6 | 14 | 8 |
| Orofacial exercise | 2 | 39 | 32 | 11 | 9 | 23 | 11 |
| Electrical stimulation | 2 | 6 | 4 | 1 | 3 | 1 | 2 |
| Electromyography (EMG) – Biofeedback therapy | 1 | 2 | 2 | 1 | 2 | 2 | 1 |
| Proprioceptive Neuromuscular Facilitation | 0 | 4 | 0 | 1 | 0 | 1 | 0 |
| Heat therapy | 1 | 3 | 1 | 0 | 1 | 1 | 1 |

Supplementary Table 6. Reasons for not using the treatments

|  | SLT |  | PT |  | Other |  | Total |  |
| --- | --- | --- | --- | --- | --- | --- | --- | --- |
|  | n=38 | % | n=41 | % | n=17 | % | n=96 | % |
|  | Response n=90 | % | Response n=77 | % | Response n=29 | % | Response n=196 | % |
| Lack of resources | 28 | 74 | 16 | 39 | 6 | 35 | 50 | 52 |
| No guidelines | 20 | 53 | 16 | 39 | 8 | 47 | 44 | 46 |
| Not confident | 22 | 58 | 18 | 44 | 8 | 47 | 48 | 50 |
| No evidence | 10 | 26 | 9 | 22 | 1 | 6 | 20 | 21 |
| No reason | 1 | 3 | 3 | 7 | 1 | 6 | 5 | 5 |
| Not applicable | 4 | 11 | 4 | 10 | 1 | 6 | 9 | 9 |
| Other | 5 | 13 | 11 | 27 | 4 | 24 | 20 | 21 |

Respondents were allowed to select more than one response. SLT: speech and language therapist; PT: physiotherapist;
