# Supplementary figures and images for: Managing Facial Palsy After Stroke: Results From an Online Survey of Health Professionals
Source: Int J Lang Commun Disord. 2025 Sep 16;60(5):e70127. doi: 10.1111/1460-6984.70127 (PMC12439456; doi:10.1111/1460-6984.70127)

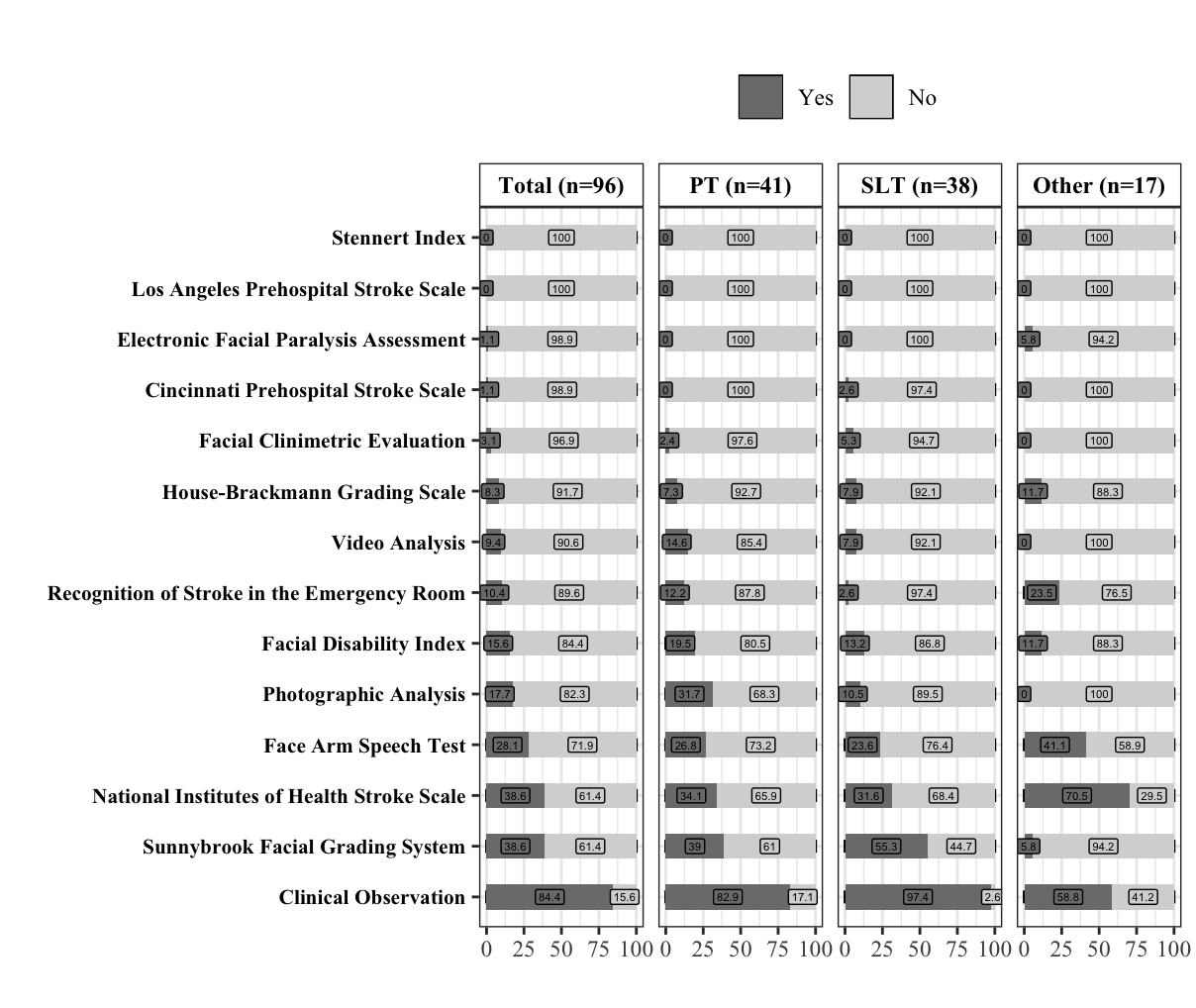

Supplement: Supplementary file 2 — Supporting: jlcd70127‐sup‐0002‐FigureS1.png [file JLCD-60-0-s002.png]

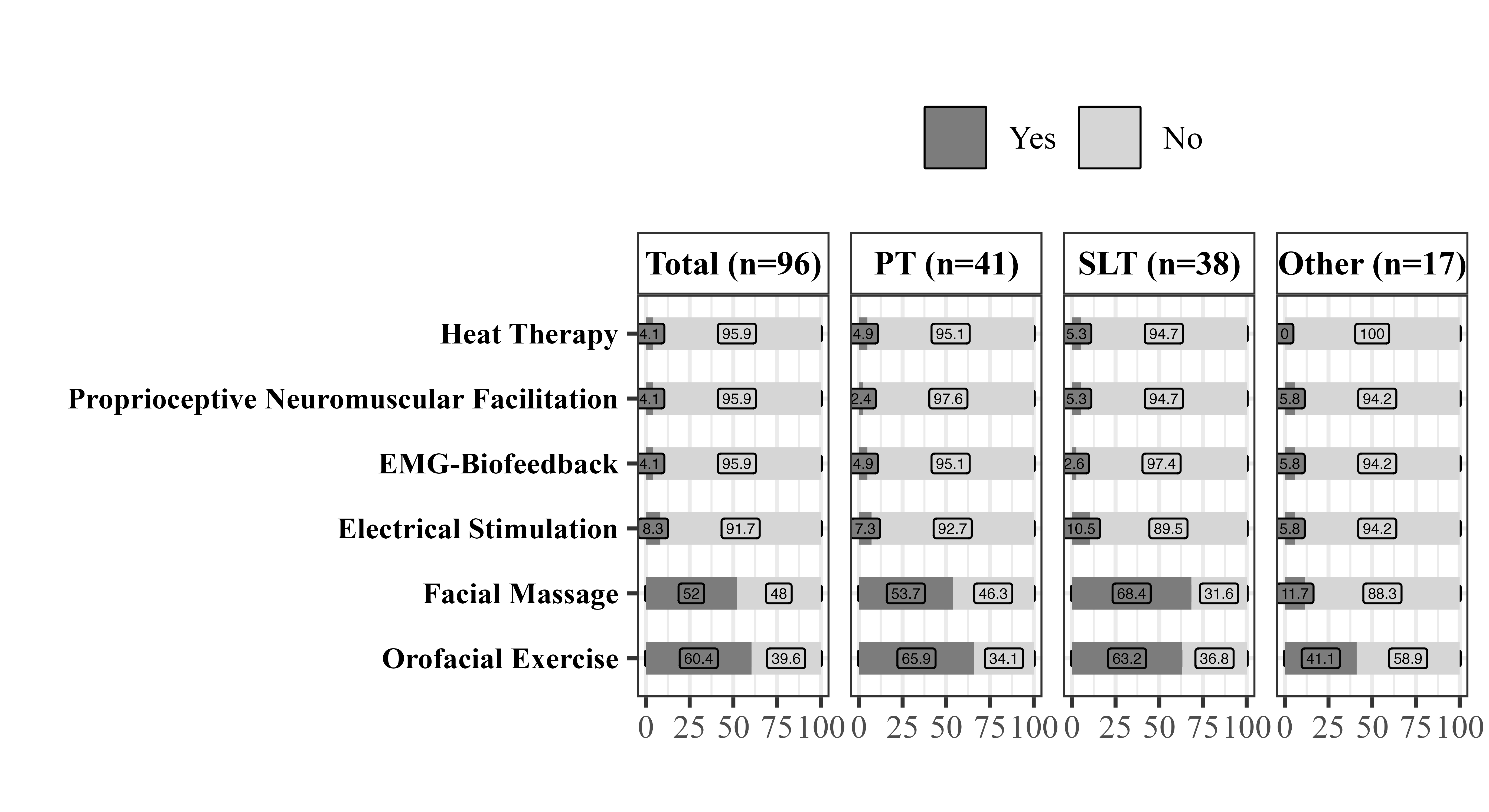

Supplement: Supplementary file 3 — Supporting: jlcd70127‐sup‐0003‐FigureS2.png [file JLCD-60-0-s003.png]
