# Supplementary material for: Managing Facial Palsy After Stroke: Results From an Online Survey of Health Professionals
Source: Int J Lang Commun Disord. 2025 Sep 16;60(5):e70127. doi: 10.1111/1460-6984.70127 (PMC12439456; doi:10.1111/1460-6984.70127)
Supplement: Supplementary file 4 — Supporting: jlcd70127‐sup‐0004‐Survey‐Questions.docx [file JLCD-60-0-s004.docx]

Supplementary Survey Questions

**An online survey for health professionals working in stroke about the management of facial palsy**

**Introduction:**

We want to understand how facial palsy after stroke is managed around the UK.

The survey consists of 3 parts. Part 1 has a few quick questions about you and your clinical service, Part 2 has some questions about how you might assess facial palsy after stroke, and Part 3 has some questions about how facial palsy after stroke might be treated.

If you wish to carry out the survey and have read the Participant Information Sheet (add link), please tick the option below. This survey is for anyone working in stroke clinical services in the UK.

- I have read the Participation Information Sheet (add link), and I consent to continue the survey.

**Part – 1:**

1. Which of the following best describes your discipline/occupation?

- Speech and Language Therapist
- Physiotherapist
- Occupational Therapist
- Stroke Nurse
- Stroke Doctor
- Maxillofacial Nurse
- Maxillofacial Doctor
- Other (please specify)

1. In which area(s) of clinical practice do you currently work? (Select all that apply)

- Acute Stroke Inpatient services
- Stroke Rehabilitation Inpatient services
- Early Supported Discharge service / Community Rehabilitation
- Long Term Community Support services
- Not applicable (please explain why not applicable)
- Other (please specify)

1. Which country do you work in?

- England
- Wales
- Scotland
- Northern Ireland
- Other (please specify)

1. How many years of experience do you have in your occupation?

- Less than one year
- 1-5 years
- 5-10 years
- Over 10 years

1. How many years have you been working with stroke patients?

- Less than one year
- 1-5 years
- 5-10 years
- Over 10 years

1. Roughly how many stroke patients are referred to your service each month?

-

1. Roughly how many stroke patients are referred to your service who have facial palsy each month?
2. Does your service get referrals specifically for Facial Palsy after stroke? (Select all that apply)

- Yes, referred specifically (If yes, continue with the 9^th^ question)
- No, picked up on the assessment (Continue with the 10^th^ question)
- Other – please explain. (Continue with the 10^th^ question)

1. Roughly how long after stroke do patients with facial palsy get referred to you?

- Less than 1 month
- 1-3 months
- 4-6 months
- 6-9 months
- 9-12 months
- Over 1 year
- Not applicable (Please comment (i.e., if you only work in early stroke care, please explain here))

1. Which healthcare professionals do you work with in the management of post-stroke facial palsy in your service? (Select all that apply)

- Speech and Language Therapist
- Physiotherapist
- Occupational Therapist
- Psychologist
- Stroke Nurse
- Stroke Doctor
- Maxillofacial Nurse
- Maxillofacial Doctor
- Not applicable
- Other (Please specify)

**Part 1 has been completed.**

**Part – 2:**

1. What kind of training have you received relating to the assessment methods to assess post-stroke facial palsy? (Select all that apply)

- Undergraduate degree
- Post-graduate degree
- Professional training (excluding the above) i.e. may or may not have led to a qualification.
- Other (please specify what kind of training)
- None

1. Do you use the National Institutes of Health Stroke Scale (NIHSS) to identify/assess facial palsy in stroke survivors?

- Yes (If yes, continue with the 13^th^ question. It's set automatically in Qualtrics)
- No (If no, continue with the 14^th^ question. It's set automatically in Qualtrics)
- Never heard of (If never heard of, continue with the 14^th^ question. It's set automatically in Qualtrics)

1. If using the National Institutes of Health Stroke Scale (NIHSS), please select the reason(s) for your selection. (Select all that apply)

- Department policy
- Professional choice
- Useful to indicate facial palsy after stroke
- Useful as part of a wider assessment
- Recommended by practice guidelines
- Widely used by others
- Other (please specify)

1. Do you use the Face Arm Speech Test (FAST) to identify/assess facial palsy in stroke survivors?

- Yes (If yes, continue with the 15^th^ question. It's set automatically in Qualtrics)
- No (If no, continue with the 16^th^ question. It's set automatically in Qualtrics)
- Never heard of (If never heard of, continue with the 16^th^ question. It's set automatically in Qualtrics)

1. If using the Face Arm Speech Test (FAST), please select the reason(s) for your selection. (Select all that apply)

- Department policy
- Professional choice
- Useful to indicate facial palsy after stroke
- Useful as part of a wider assessment
- Recommended by practice guidelines
- Widely used by others
- Other (please specify)

1. Do you use the Cincinnati Prehospital Stroke Scale (CPSS) to identify/assess facial palsy in stroke survivors?

- Yes (If yes, continue with the 17^th^ question. It's set automatically in Qualtrics)
- No (If no, continue with the 18^th^ question. It's set automatically in Qualtrics)
- Never heard of (If never heard of, continue with the 18^th^ question. It's set automatically in Qualtrics)

1. If using the Cincinnati Prehospital Stroke Scale (CPSS), please select the reason(s) for your selection. (Select all that apply)

- Department policy
- Professional choice
- Useful to indicate facial palsy after stroke
- Useful as part of a wider assessment
- Recommended by practice guidelines
- Widely used by others
- Other (please specify)

1. Do you use Los Angeles Prehospital Stroke Scale (LAPSS) to identify/assess facial palsy in stroke survivors?

- Yes (If yes, continue with the 19^th^ question. It's set automatically in Qualtrics)
- No (If no, continue with the 20^th^ question. It's set automatically in Qualtrics)
- Never heard of (If never heard of, continue with the 20^th^ question. It's set automatically in Qualtrics)

1. If using the Los Angeles Prehospital Stroke Scale (LAPSS), please select the reason(s) for your selection. (Select all that apply)

- Department policy
- Professional choice
- Useful to indicate facial palsy after stroke
- Useful as part of a wider assessment
- Recommended by practice guidelines
- Widely used by others
- Other (please specify)

1. Do you use the Recognition of Stroke in the Emergency Room (ROSIER) scale to identify/assess facial palsy in stroke survivors?

- Yes (If yes, continue with the 21^st^ question. It's set automatically in Qualtrics)
- No (If no, continue with the 22^nd^ question. It's set automatically in Qualtrics)
- Never heard of (If never heard of, continue with the 22^nd^ question. It's set automatically in Qualtrics)

1. If using the Recognition of Stroke in the Emergency Room (ROSIER), please select the reason(s) for your selection. (Select all that apply)

- Department policy
- Professional choice
- Useful to indicate facial palsy after stroke
- Useful as part of a wider assessment
- Recommended by practice guidelines
- Widely used by others
- Other (please specify)

1. Do you use the Stennert Index to identify/assess facial palsy in stroke survivors?

- Yes (If yes, continue with the 23^rd^ question. It's set automatically in Qualtrics)
- No (If no, continue with the 24^th^ question. It's set automatically in Qualtrics)
- Never heard of (If never heard of, continue with the 24^st^ question. It's set automatically in Qualtrics)

1. If using the Stennert Index, please select the reason(s) for your selection. (Select all that apply)

- Department policy
- Professional choice
- Useful to indicate facial palsy after stroke
- Useful as part of a wider assessment
- Recommended by practice guidelines
- Widely used by others
- Other (please specify)

1. Do you use the Facial Disability Index (FDI) to identify/assess facial palsy in stroke survivors?

- Yes (If yes, continue with the 25^th^ question. It's set automatically in Qualtrics)
- No (If no, continue with the 26^th^ question. It's set automatically in Qualtrics)
- Never heard of (If never heard of, continue with the 26^th^ question. It's set automatically in Qualtrics)
- Not applicable (If not applicable, continue with the 26^th^ question. It's set automatically in Qualtrics)

1. If using the Facial Disability Index (FDI), please select the reason(s) for your selection. (Select all that apply)

- Department policy
- Professional choice
- Useful to indicate facial palsy after stroke
- Useful as part of a wider assessment
- Recommended by practice guidelines
- Widely used by others
- Other (please specify)

1. Do you use The Facial Clinimetric Evaluation Scale (FaCE) to identify/assess facial palsy in stroke survivors?

- Yes (If yes, continue with the 27^th^ question. It's set automatically in Qualtrics)
- No (If no, continue with the 28^th^ question. It's set automatically in Qualtrics)
- Never heard of (If never heard of, continue with the 28^th^ question. It's set automatically in Qualtrics)

1. If using The Facial Clinimetric Evaluation Scale (FaCE), please select the reason(s) for your selection. (Select all that apply)

- Department policy
- Professional choice
- Useful to indicate facial palsy after stroke
- Useful as part of a wider assessment
- Recommended by practice guidelines
- Widely used by others
- Other (please specify)

1. Do you use the House-Brackmann Facial Nerve Grading System (HBGS) to identify/assess facial palsy in stroke survivors?

- Yes (If yes, continue with the 29^th^ question. It's set automatically in Qualtrics)
- No (If no, continue with the 30^th^ question. It's set automatically in Qualtrics)
- Never heard of (If never heard of, continue with the 30^th^ question. It's set automatically in Qualtrics)

1. If using the House-Brackmann Facial Nerve Grading System (HBGS), please select the reason(s) for your selection. (Select all that apply)

- Department policy
- Professional choice
- Useful to indicate facial palsy after stroke
- Useful as part of a wider assessment
- Recommended by practice guidelines
- Widely used by others
- Other (please specify)

1. Do you use Sunnybrook Facial Grading System to identify/assess facial palsy in stroke survivors?

- Yes (If yes, continue with the 31^st^ question. It's set automatically in Qualtrics)
- No (If no, continue with the 32^nd^ question. It's set automatically in Qualtrics)
- Never heard of (If never heard of, continue with the 32^nd^ question. It's set automatically in Qualtrics)

1. If using Sunnybrook Facial Grading System, please select the reason(s) for your selection. (Select all that apply)

- Department policy
- Professional choice
- Useful to indicate facial palsy after stroke
- Useful as part of a wider assessment
- Recommended by practice guidelines
- Widely used by others
- Other (please specify)

1. Do you use the electronic facial paralysis assessment tool (eFACE) to identify/assess facial palsy in stroke survivors?

- Yes (If yes, continue with the 33^rd^ question. It's set automatically in Qualtrics)
- No (If no, continue with the 34^th^ question. It's set automatically in Qualtrics)
- Never heard of (If never heard of, continue with the 34^th^ question. It's set automatically in Qualtrics)

1. If using the electronic facial paralysis assessment tool (eFACE), please select the reason(s) for your selection. (Select all that apply)

- Department policy
- Professional choice
- Useful to indicate facial palsy after stroke
- Useful as part of a wider assessment
- Recommended by practice guidelines
- Widely used by others
- Other (please specify)

1. Do you use the photographic analysis to identify/assess facial palsy in stroke survivors?

- Yes (If yes, continue with the 35^th^ question. It's set automatically in Qualtrics)
- No (If no, continue with the 36^th^ question. It's set automatically in Qualtrics)
- Never heard of (If never heard of, continue with the 36^th^ question. It's set automatically in Qualtrics)

1. If using the photographic analysis, please select the reason(s) for your selection. (Select all that apply)

- Department policy
- Professional choice
- Useful to indicate facial palsy after stroke
- Useful as part of a wider assessment
- Recommended by practice guidelines
- Widely used by others
- Other (please specify)

1. Do you use the Video analysis (2D, 3D) to identify/assess facial palsy in stroke survivors?

- Yes (If yes, continue with the 37^th^ question. It's set automatically in Qualtrics)
- No (If no, continue with the 38^th^ question. It's set automatically in Qualtrics)
- Never heard of (If never heard of, continue with the 38^th^ question. It's set automatically in Qualtrics)

1. If using the Video analysis (2D, 3D), please select the reason(s) for your selection. (Select all that apply)

- Department policy
- Professional choice
- Useful to indicate facial palsy after stroke
- Useful as part of a wider assessment
- Recommended by practice guidelines
- Widely used by others
- Other (please specify)

1. Do you use Clinical Observation methods (informal or structured) to identify/assess facial palsy in stroke survivors?

- Yes (If yes, continue with the 39^th^ question. It's set automatically in Qualtrics)
- No (If no, continue with the 40^th^ question. It's set automatically in Qualtrics)
- Never heard of (If never heard of, continue with the 40^th^ question. It's set automatically in Qualtrics)

1. If using the Clinical Observation methods (informal or structured), please select the reason(s) for your selection. (Select all that apply)

- Department policy
- Professional choice
- Useful to indicate facial palsy after stroke
- Useful as part of a wider assessment
- Recommended by practice guidelines
- Widely used by others
- Other (please specify)

1. If you do not use any of the assessments asked in the previous questions, would you please choose the reasons? (Select all that apply)

- Lack of resources
- No guidelines
- Not confident
- No evidence
- No reason
- Not applicable
- Other (please specify why you don’t use)

1. Does your stroke service use any methods, not described previously, to identify/assess facial palsy?

- Yes (please expand on your answer by adding a comment)
- No (You can add a comment if you want, but you don't have to add anything to continue)

**Part 2 has been completed. Part 3 is similar to Part 2 but with fewer questions.**

Part – 3:

1. What kind of training have you received relating to the treatment methods of post-stroke facial palsy? (Select all that apply)

- Undergraduate degree
- Post-graduate degree
- Professional training (excluding the above) i.e. may or may not have led to a qualification.
- Other (please specify what kind of training)
- None

1. Do you use facial massage as a treatment for stroke survivors with facial palsy?

- Yes (If yes, continue with the 44^th^ question. It's set automatically in Qualtrics)
- No (If no, continue with the 45^th^ question. It's set automatically in Qualtrics)
- Never heard of (If never heard of, continue with the 45^th^ question. It's set automatically in Qualtrics)

1. If using facial massage, please select the reason(s) for your selection. (Select all that apply)

- Department policy
- Professional choice
- Useful to indicate facial palsy after stroke
- Useful as part of a wider assessment
- Recommended by practice guidelines
- Widely used by others
- Other (please specify)

1. Do you use orofacial exercise as a treatment for stroke survivors with facial palsy?

- Yes (If yes, continue with the 46^th^ question. It's set automatically in Qualtrics)
- No (If no, continue with the 47^th^ question. It's set automatically in Qualtrics)
- Never heard of (If never heard of, continue with the 47^th^ question. It's set automatically in Qualtrics)

1. If using orofacial exercise, please select the reason(s) for your selection. (Select all that apply)

- Department policy
- Professional choice
- Useful to indicate facial palsy after stroke
- Useful as part of a wider assessment
- Recommended by practice guidelines
- Widely used by others
- Other (please specify)

1. Do you use electrical stimulation as a treatment for stroke survivors with facial palsy?

- Yes (If yes, continue with the 48^th^ question and then 49^th^ question. It's set automatically in Qualtrics)
- No (If no, continue with the 50^th^ question. It's set automatically in Qualtrics)
- Never heard of (If never heard of, continue with the 50^th^ question. It's set automatically in Qualtrics)

1. If using electrical stimulation, please select the reason(s) for your selection. (Select all that apply)

- Department policy
- Professional choice
- Useful to indicate facial palsy after stroke
- Useful as part of a wider assessment
- Recommended by practice guidelines
- Widely used by others
- Other (please specify)

1. What sort of electrical stimulation do you use for stroke survivors with facial palsy? (Could you also specify which brand it is?)
2. Do you use Electromyography (EMG) – Biofeedback therapy as a treatment for stroke survivors with facial palsy?

- Yes (If yes, continue with the 51^st^ question. It's set automatically in Qualtrics)
- No (If no, continue with the 52^nd^ question. It's set automatically in Qualtrics)
- Never heard of (If never heard of, continue with the 52^nd^ question. It's set automatically in Qualtrics)

1. If using Electromyography (EMG) – Biofeedback therapy, please select the reason(s) for your selection. (Select all that apply)

- Department policy
- Professional choice
- Useful to indicate facial palsy after stroke
- Useful as part of a wider assessment
- Recommended by practice guidelines
- Widely used by others
- Other (please specify)

1. Do you use Proprioceptive Neuromuscular Facilitation (PNF) as a treatment for stroke survivors with facial palsy?

- Yes (If yes, continue with the 53^rd^ question. It's set automatically in Qualtrics)
- No (If no, continue with the 54^th^ question. It's set automatically in Qualtrics)
- Never heard of (If never heard of, continue with the 54^th^ question. It's set automatically in Qualtrics)

1. If using Proprioceptive Neuromuscular Facilitation (PNF), please select the reason(s) for your selection. (Select all that apply)

- Department policy
- Professional choice
- Useful to indicate facial palsy after stroke
- Useful as part of a wider assessment
- Recommended by practice guidelines
- Widely used by others
- Other (please specify)

1. Do you use heat therapy as a treatment for stroke survivors with facial palsy?

- Yes (If yes, continue with the 55^th^ question. It's set automatically in Qualtrics)
- No (If no, continue with the 56^th^ question. It's set automatically in Qualtrics)
- Never heard of (If never heard of, continue with the 56^th^ question. It's set automatically in Qualtrics)

1. If using heat therapy, please select the reason(s) for your selection. (Select all that apply)

- Department policy
- Professional choice
- Useful to indicate facial palsy after stroke
- Useful as part of a wider assessment
- Recommended by practice guidelines
- Widely used by others
- Other (please specify)

1. If you do not use any of the treatments asked in the previous questions, would you please choose the reasons? (Select all that apply)

- Lack of resources
- No guidelines
- Not confident
- No evidence
- No reason
- Not applicable
- Other (please specify why you don’t use)

1. Does your stroke service use any methods, not described previously, to treat facial palsy?

- Yes (please expand on your answer by adding a comment)
- No (You can add a comment if you want, but you don't have to add anything to continue)

**We thank you for your time spent taking this survey.**

**Your response has been recorded.**

**If you have any questions or are interested in joining a mailing list to receive updates on this study's findings or to find out about other research around facial palsy, then please do not hesitate to e-mail (havvasumeyye.eroglu@postgrad.manchester.ac.uk)**
